# Advanced dose calculation strategies for clinical linear accelerators: a systematic review

**DOI:** 10.3389/fonc.2026.1790425

**Published:** 2026-05-01

**Authors:** Ali H. D. Alshehri, Abdulrahman Al Mopti

**Affiliations:** 1 Department of Radiological Sciences, College of Applied Medical Sciences, Najran University, Najran, Saudi Arabia; 2 Health Research Center, Najran University, Najran, Saudi Arabia

**Keywords:** adaptive radiotherapy, artificial intelligence, clinical linear accelerators, dose calculation, GPU acceleration, high-precision radiotherapy, LINAC dosimetry, Monte Carlo simulation

## Abstract

**Background:**

Delivering accurate radiation doses in heterogeneous tissues is critical in radiotherapy, yet conventional algorithms often lose accuracy in complex scenarios. Monte Carlo (MC) simulation offers a high-fidelity approach, but routine clinical use has historically been limited by computation time.

**Purpose:**

This systematic review evaluates the clinical relevance of advanced MC-based dose-calculation strategies for linear accelerators (LINACs), with emphasis on developments from 2010 to 2025 that improve dose accuracy and workflow efficiency, particularly through GPU acceleration and artificial intelligence (AI).

**Methods:**

Following the PRISMA 2020 guideline, PubMed, Scopus, and Web of Science were searched. The search identified 467 records; after deduplication, 312 were screened, and 17 eligible studies were included across Varian-, Elekta-, and Siemens-associated platforms. Data were extracted on simulation techniques, clinical application, and performance outcomes. Study quality was appraised using a predefined four-domain framework informed by the AAPM TG-268 RECORDS checklist, assessing methodological rigor, validation completeness, clinical relevance, and uncertainty analysis. Data synthesis followed the Synthesis Without Meta-analysis (SWiM) guidance.

**Results:**

MC-based dose calculations consistently outperformed or matched conventional algorithms in small-field, heterogeneity-rich, and magnetic-field scenarios. GPU implementations achieved 50--2500x speed improvements with less than 1% reported dose deviation. AI applications were used mainly to reduce noise and computation time. Elekta’s Monaco TPS includes a clinically validated fast MC engine, whereas Varian-associated workflows more commonly use MC for independent quality assurance. Studies involving the Elekta Unity MR-Linac confirmed accurate modeling of magnetic-field effects. Only one included study addressed a Siemens LINAC platform.

**Conclusions:**

Accelerated MC strategies now permit accurate and efficient dose calculation that may support routine clinical workflows. However, direct evidence linking these dosimetric gains to improved clinical outcomes remains limited.

**Systematic review registration:**

https://osf.io/ftnbs, identifier 10.17605/OSF.IO/FTNBS.

## 1 Introduction

Cancer remains one of the leading causes of mortality worldwide, with an estimated 20.0 million new cases and 9.7 million deaths in 2022 (1). Radiotherapy is a cornerstone of cancer treatment and has undergone substantial technological development aimed at improving precision. Modern techniques such as intensity-modulated radiotherapy (IMRT) and image-guided radiotherapy (IGRT) allow high radiation doses to be delivered to tumors while better sparing adjacent healthy tissue (2). For example, daily IGRT imaging can be used to adjust patient positioning or dose, improving targeting accuracy and reducing normal-tissue exposure (2). Linear accelerators (LINACs) are the principal devices used in external beam radiotherapy, producing high-energy photon and electron beams for deep-seated tumors. In clinical practice, photon beam energies are typically specified in megavolts (MV), referring to the LINAC accelerating potential. Over the past decade, hybrid systems such as magnetic resonance-guided LINACs (MR-Linacs) have emerged, combining real-time MR imaging with radiation delivery to support adaptive radiotherapy based on daily patient anatomy. These developments support increasingly individualized radiotherapy workflows.

A central dosimetric challenge in radiotherapy is accurate dose calculation in heterogeneous tissues such as lung, bone, or regions with air cavities. At density interfaces, conventional analytical algorithms (e.g., pencil-beam and convolution/superposition methods) can lose accuracy. For instance, the widely used Analytical Anisotropic Algorithm (AAA) shows limitations in lung and bone regions (3). Varian’s Acuros XB algorithm, a deterministic Boltzmann transport solver, achieves better agreement with Monte Carlo in such heterogeneous media (4). While algorithms such as Acuros XB and collapsed-cone convolution run faster than MC, the Monte Carlo method is generally regarded as the reference standard for dose calculation because it models particle interactions in detail (5, 6). MC simulations track millions of individual photons and electrons through patient or phantom geometry, statistically accounting for scattering and absorption with minimal simplifying assumptions. This accuracy is important because even a systematic dose deviation on the order of 5% can affect tumor control and normal tissue complication probabilities (7). These considerations provide a strong rationale for using the most accurate dose engines available for treatment planning and verification.

In this context, Monte Carlo simulation is widely used for commissioning LINAC beam models, verifying treatment plan doses, and evaluating new treatment techniques. However, despite its superior accuracy, conventional MC has historically been too computationally intensive for routine planning and has therefore often been confined to offline studies or quality assurance workflows. Over the past 15 years, substantial effort has been directed toward reducing this limitation. GPU acceleration and variance-reduction techniques have markedly shortened computation times (8). More recently, AI-assisted approaches, such as surrogate models and dose-denoising networks, have been investigated to further accelerate MC-based workflows (9). Collectively, these developments have improved the clinical feasibility of MC dose calculation.

This systematic review focuses on the clinical applicability of MC dose calculation rather than its accuracy in isolation. The review maps platform-specific MC implementations across major vendors, identifies the clinical scenarios in which MC corrections are large enough to affect treatment decisions, distinguishes these from scenarios in which the corrections are dosimetrically measurable but clinically inconsequential, and reports the practical barriers that remain after computation time is no longer limiting. Vendor-specific search terms (Varian, Elekta, Siemens) were included to enable direct mapping of platform-specific MC implementations. The review evaluates where and how MC methods improve dose calculation relative to conventional algorithms and how recent advances in GPU computing and AI may support routine clinical implementation, particularly for advanced techniques such as stereotactic radiosurgery (SRS) and adaptive radiotherapy.

Elekta’s Monaco treatment planning system employs a built-in clinical Monte Carlo dose engine, which has been independently commissioned and validated on Elekta Versa HD LINACs (10, 11). These developments indicate that fully three-dimensional Monte Carlo dose calculation, once too slow for typical clinical timelines, can now be implemented within selected radiotherapy workflows.

The review protocol was registered on the Open Science Framework (OSF) Registries (DOI: 10.17605/OSF.IO/FTNBS; 12) to document the review methods and ensure methodological transparency. This review is reported following the PRISMA 2020 guidelines (13), and data synthesis follows the Synthesis Without Meta-analysis (SWiM) reporting guideline (14).

## 2 Methods

### 2.1 Literature search and study selection

A systematic literature search was conducted to identify relevant peer-reviewed studies published from 2010 through September 3, 2025. PubMed, Scopus, and Web of Science were queried on that date using the combined keywords:

*(“Monte Carlo” OR MC) AND (“linear accelerator” OR LINAC) AND (“radiotherapy” OR “dosimetry”) AND (“Varian” OR “Elekta” OR “Siemens”) AND (“GPU” OR “accelerated” OR “artificial intelligence” OR AI)*.

Vendor terms (Varian, Elekta, Siemens) were included to enable mapping and comparison of MC implementations across the major clinical LINAC platforms. This strategy may have excluded MC studies that did not explicitly name a vendor in the title, abstract, or keywords. To partially mitigate this limitation, manual backward citation screening of reference lists from key review articles was also performed (5, 9, 15). The search was limited to English-language publications.

**Figure 1:**
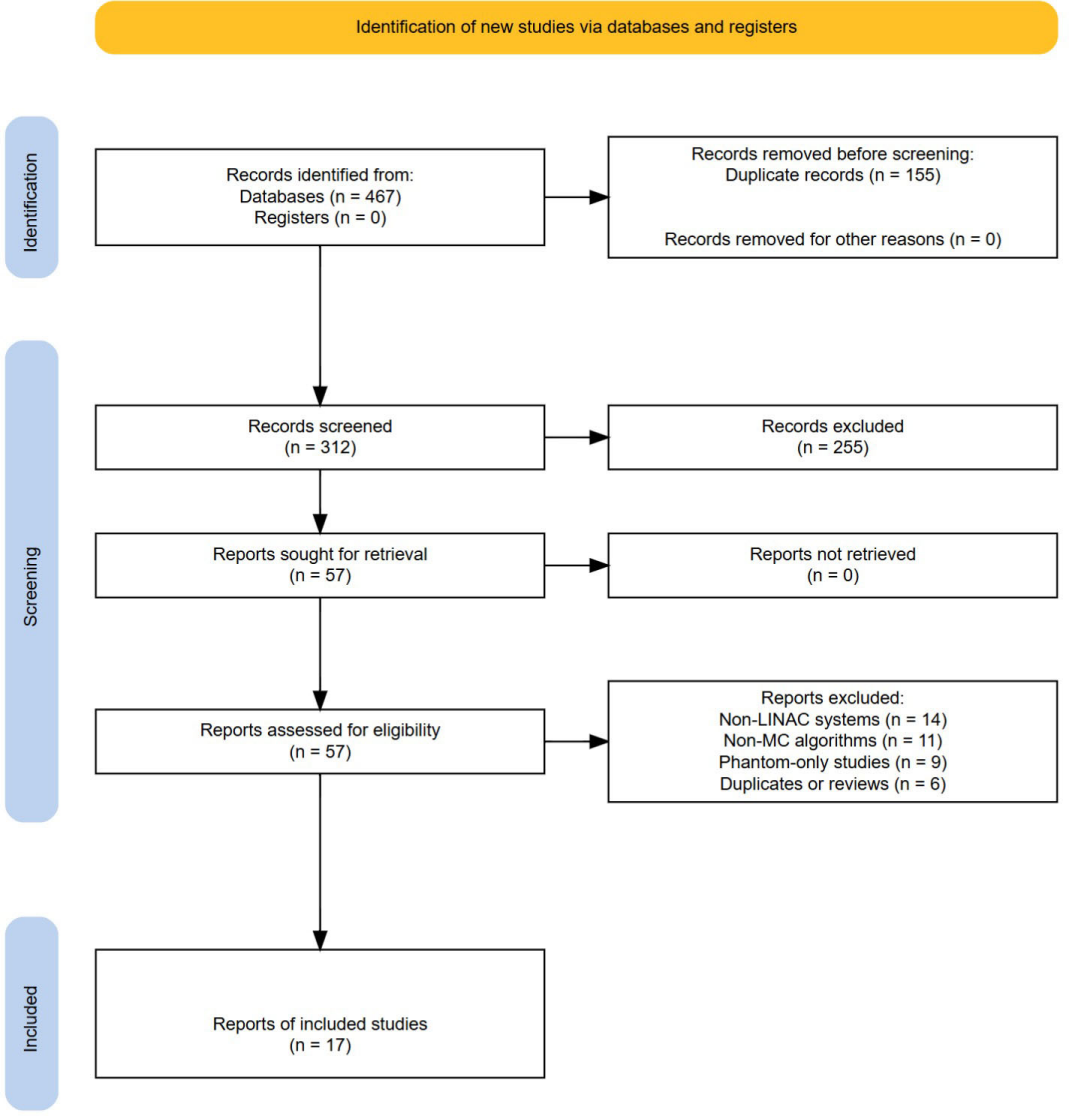
PRISMA 2020 flow diagram of study selection.

The search yielded 467 records across the three databases. After duplicate removal, 312 unique records remained. Titles and abstracts were screened independently by two reviewers, and disagreements were resolved through discussion until consensus was reached. At this stage, 255 records were excluded, leaving 57 articles for full-text evaluation. Full-text articles were then assessed independently by two reviewers against the inclusion and exclusion criteria, with disagreements again resolved through discussion. Forty articles were excluded for the following reasons: non-LINAC systems (n = 14), non-MC algorithms (n = 11), phantom-only studies without clinical relevance (n = 9), and duplicates or review articles (n = 6). A total of 17 studies met the inclusion criteria for detailed analysis. **Figure 1** outlines the study selection process (PRISMA 2020 flow diagram).

#### 2.1.1 Inclusion criteria

Peer-reviewed studies published between 2010 and September 2025 that investigated photon- or electron-beam Monte Carlo dose simulation for clinical LINACs. Eligible studies included experimental validation of MC models and/or comparisons with treatment planning system (TPS) calculations, as well as studies presenting methods to accelerate MC for clinical use, including GPU implementations or AI-based speed-up and denoising strategies applied to LINAC dose simulation. Prospective studies, retrospective studies, and technical development papers were included provided that new data or methods were presented in a clinical radiotherapy context.

#### 2.1.2 Exclusion criteria

Studies on particle therapy (proton or heavy-ion beams) or brachytherapy were excluded because the review focused on external photon- and electron-beam LINACs. Studies that did not involve clinical LINAC dosimetry were also excluded, for example purely methodological MC papers without validation on actual LINAC beams or patient treatments. Review articles were excluded from the primary analysis to avoid summarizing secondary literature; however, key reviews were retained as contextual references. Other exclusions were non-English articles, conference abstracts without full papers, preprints or white papers, and duplicate publications.

No meta-analysis was planned because the included outcomes and methodologies were too heterogeneous for quantitative pooling. Data synthesis was conducted according to the Synthesis Without Meta-analysis (SWiM) reporting guideline (14), and PRISMA 2020 guidance was followed for reporting the search and study-selection process (13). For comparative studies, synthesis also included vote counting by direction of effect in line with SWiM and Cochrane guidance.

### 2.2 Data extraction and quality assessment

For each of the 17 included studies, 11 predefined entities were extracted using a standardized data-charting form: (1) study identification (authors, year, journal); (2) LINAC vendor and model; (3) MC code and version; (4) study objective; (5) beam characteristics; (6) acceleration technique and GPU hardware specifications; (7) computational performance metrics; (8) dosimetric outcomes; (9) validation method; (10) clinical application context, including dose-reporting convention; and (11) quality assessment rating. Initial extraction was performed by one reviewer and independently checked by a second reviewer for accuracy and completeness.

Methodological quality was appraised using a predefined framework informed by the AAPM Task Group 268 RECORDS checklist for reporting Monte Carlo radiation transport studies (16), the AHRQ Guidance for Modeling and Simulation Studies (17), and the JBI Critical Appraisal Checklist for Analytical Cross-Sectional Studies (18), adapted for technical simulation research. Standard risk-of-bias tools (e.g., ROBIS, AMSTAR-2) are intended for systematic reviews of clinical interventions and were not considered directly applicable to simulation and dosimetry studies. Each study was assessed across four domains: (1) methodological rigor, including clarity of MC code identification, geometry modeling, physics settings, variance-reduction techniques, and source model characterization; (2) validation completeness, including independent measurements, detector types, setup conditions, multiple configurations tested, and appropriate agreement metrics; (3) clinical relevance, including use of clinical beam energies and realistic geometries; and (4) uncertainty analysis, including reported statistical uncertainties, convergence testing, and sensitivity analyzes. Each domain was rated as satisfactory or unsatisfactory. Overall quality was rated High (4/4 domains satisfactory), Moderate (3/4), or Low (2 or fewer). Two reviewers assessed quality independently, and disagreements were resolved through discussion. Quality ratings informed interpretation of the evidence but did not determine study exclusion.

#### 2.2.1 Bias and limitations

Because the included literature consisted primarily of simulation and experimental dosimetry studies rather than clinical outcome trials, traditional risk-of-bias tools were not directly applicable. Instead, the quality assessment focused on potential technical biases and methodological limitations within each study. Review-process bias was minimized through independent study selection, quality appraisal, and the use of prespecified review methods documented in the OSF protocol. The vendor-specific search strategy may nevertheless have introduced selection bias by excluding MC studies that did not name a commercial LINAC platform explicitly; this limitation should be considered when interpreting the completeness of coverage.

## 3 Results

### 3.1 Study selection and characteristics

Seventeen studies, published between 2010 and 2025, were included in this review. **Table 1** summarizes the main characteristics of the included studies, including platform, study objective, acceleration approach, key dosimetric findings, and quality rating. Most studies focused on photon-beam dose calculation and validation for clinical LINAC workflows, with comparisons against measured data, conventional TPS algorithms, or both. The distribution of the 17 included studies across LINAC vendor and platform categories is summarized in **Table 2**.

**Table 1:** Characteristics of the included studies evaluating Monte Carlo dose calculation for clinical linear accelerators.

Study (year)	Platform/vendor	Focus/objective	Acceleration	Key findings/contributions	Quality
Hillman et al. (19)	Varian/Edge (6X FFF, HD-MLC)	Refinement of Mobius3D (M3D) beam model for SRS/SBRT patient-specific QA	None	Refined HD-MLC beam model improved mean gamma passing rate from 94.6% to 98.0% for SRS/SBRT cases; highlighted the importance of small-field MLC modeling for QA accuracy	High
Menon et al. (20)	Varian/Novalis TX	Dose algorithm comparison in AVM stereotactic radiosurgery	None	MC outperformed pencil-beam calculations in small targets and was recommended for intracranial SRS planning.	High
Zope et al. (21)	Varian/Unique Performance (6 MV)	Photon beam commissioning and RapidArc prerequisite QA	None	Reported commissioning measurements (PDD, profiles, MLC transmission, flatness, symmetry) for a low-energy Varian Unique Performance LINAC; complemented by RapidArc QA procedures	Low
Bergman et al. (22)	Varian/TrueBeam	HD120 MLC modeling and SABR plan verification	None	MC reproduced HD120 MLC dosimetry with agreement within approximately 2% of measurement.	Moderate
Han et al. (23)	Varian/Eclipse TPS	Acuros XB versus MC in heterogeneous media	None	Acuros XB agreed within approximately 2% of MC; MC was treated as the reference standard.	Moderate
Hissoiny et al. (8)	Vendor-independent platform	Development of GPUMCD	GPU	Reported more than 100x acceleration with less than 1% dose error, supporting early clinical GPU-MC use.	High
Jia et al. (24)	Vendor-independent platform	GPU-based MC for IMRT dose calculation	GPU	Reported approximately 50x speedup, patient-plan dose calculation in less than 1 minute, and less than 1% error.	High
Liu et al. (25)	Vendor-independent platform	GARDEN engine for fast MC dose calculation	GPU	Reported approximately 2500x acceleration over Geant4 with less than 1% error and approximately 3-second dose calculation.	High
Sadrollahi et al. (26)	Siemens/Artiste	6 MV and 7 MV FFF beam dosimetry	None	MC reproduced measured beam profiles with less than 2% deviation.	Moderate
Snyder et al. (10)	Elekta/Versa HD	Commissioning of the Monaco MC dose engine	None	Clinical commissioning results supported the validity of the Monaco MC algorithm on Versa HD.	Moderate
Kolacio et al. (11)	Elekta/Versa HD	Validation of Monaco MC options with MCNP	None	Independent MCNP calculations supported validation of Monaco MC dose options.	Moderate
Renil Mon et al. (27)	Elekta/Synergy	SRS cone modeling and validation	None	MC dose calculations matched film measurements within approximately 1% for cone-based fields.	High
Friedel et al. (28)	Elekta/Unity MR-Linac	Full MC modeling of the 1.5 T MR-Linac	None	Reported 99.8% gamma agreement (3%/3 mm) with the vendor TPS.	High
Yano et al. (29)	Elekta/Unity MR-Linac	Small-field dosimetry in a magnetic field	None	MC quantified magnetic-field effects and provided output-factor correction data.	Moderate
Margaroni et al. (30)	Elekta/Unity MR-Linac	Chamber correction and air-gap dosimetry	None	MC-derived kBQ factors and air-gap correction factors were reported for the Unity platform.	High
Cheng et al. (31)	Elekta/Unity MR-Linac	Online adaptive plan verification using ArcherQA	GPU	Reported approximately 99% gamma pass rates with dose recalculation completed in about 1–2 minutes.	High
Krishnan and Momeen (32)	Elekta/Monaco TPS	Influence of reference selection on gamma analysis	None	Gamma pass rates differed according to whether MC or measurement was used as the reference.	Low

AVM, arteriovenous malformation; FFF, flattening-filter-free; IMRT, intensity-modulated radiotherapy; MC, Monte Carlo; MLC, multileaf collimator; SABR, stereotactic ablative radiotherapy; SRS, stereotactic radiosurgery; TPS, treatment planning system.

**Table 2:** Distribution of included studies by LINAC vendor or platform type.

Vendor/platform type	Studies (n)	Share (%)
Varian-associated studies	5	29.4
Elekta-associated studies	8	47.1
Siemens-associated studies	1	5.9
Vendor-independent MC platforms	3	17.6

Vendor-associated categories were based on the primary platform evaluated in each included study.

### 3.2 Monte Carlo accuracy across LINAC platforms

#### 3.2.1 Varian LINACs

Monte Carlo simulations have been applied to Varian-associated platforms (e.g., TrueBeam, Novalis TX, Eclipse-associated workflows, and lower-energy Unique Performance LINACs; 21). MC improved dose agreement in small-field SRS by 3-8% compared with pencil-beam algorithms in heterogeneous regions (20). For TrueBeam HD120 MLC modeling, MC-calculated doses agreed within 2% of measurements for SABR plans (22). Han et al. (23) showed that Acuros XB agreed within 2% of MC in heterogeneous media, supporting the use of MC as a reference standard. In Eclipse-based clinical environments, MC therefore remains most relevant as an independent verification or commissioning tool rather than as the native treatment-planning dose engine (23). GPU acceleration has markedly reduced runtime: early GPU-based codes such as GPUMCD and gPMC achieved approximately 50-100x speedups (8, 24), and Liu et al. (25) reported a GPU MC engine with less than 1% error and approximately 2500x acceleration. More broadly, vendor-independent GPU-MC developments have improved the feasibility of applying MC within selected clinical workflows.

#### 3.2.2 Elekta LINACs

Elekta systems, including the Monaco TPS and the Unity 1.5 T MR-Linac, have been emphasized in recent MC studies. Monaco’s native MC dose engine has been commissioned and validated (e.g., 10, 11). Conventional Elekta beams have also been modeled: Renil Mon et al. (27) showed that MC dose calculations for small SRS cones matched film measurements within approximately 1%. In MR-guided radiotherapy, MC remains particularly important. Friedel et al. (28) developed a comprehensive MC model of the Unity, including the 1.5 T magnetic field, and reported 99.8% gamma agreement (3%/3 mm) with the vendor TPS. Yano et al. (29) used MC to quantify magnetic-field effects on Unity small fields and derived output-factor corrections. Margaroni et al. (30) derived ion-chamber calibration factors for the Unity using MC. To support online adaptive therapy, Cheng et al. (31) reported a GPU-based MC module (ArcherQA) that recalculated adapted Unity plans in approximately 1–2 minutes with about 99% gamma pass rates. Taken together, these findings indicate that MC can model Elekta systems accurately and can support time-sensitive QA workflows.

Elekta-associated studies formed the largest subgroup in the review, reflecting two structural factors: Monaco is the only widely used commercial TPS with a native clinical MC dose engine, and the Unity MR-Linac has generated substantial MC research because of the dosimetric challenges introduced by the 1.5 T magnetic field.

#### 3.2.3 Siemens LINACs

Although Siemens exited the LINAC market, existing machines have been modeled. Sadrollahi et al. (26) modeled a Siemens Artiste 6 MV and 7 MV FFF beam using Geant4, reproducing measurements within approximately 2%. This finding indicates that Monte Carlo-based beam modeling remains feasible for Siemens-associated LINAC platforms despite the limited vendor-specific evidence.

### 3.3 GPU acceleration: from research tool to clinical enabler

GPU acceleration has been a major technological driver of clinical MC implementation. **Table 3** summarizes selected GPU-accelerated MC dose engines relevant to the reviewed literature and compares their design features, reported advantages, and current limitations. Reported computation times should nevertheless be interpreted in light of GPU architecture, memory bandwidth, and CUDA-core availability.

**Table 3:** Selected GPU-accelerated Monte Carlo dose engines relevant to the reviewed literature.

Code name	Original basis	Key features	Advantages	Limitations	Reference
GPUMCD	Independent GPU implementation	Early clinical GPU-MC dose calculator used in QA workflows	More than 100x faster than CPU implementations; early clinical applicability	Closed-source and primarily QA-oriented	Hissoiny et al. (8)
gPMC	PENELOPE-derived GPU adaptation	GPU implementation for photon dose calculation in patient CT data	Approximately 50-60x speedup with support for patient-specific calculations	Limited to photon transport and mainly reported as a research tool	Jia et al. (24)
FRED	Independent CUDA/C++ code	General-purpose GPU MC for electrons and photons	Open-source; supports a wide energy range including VHEE applications	Fewer clinical validation studies than older platforms	Franciosini et al. (33)
gDPM	Derived from DPM/EGS-based work	Extended for Elekta Unity 1.5 T MR-Linac dose calculation	Fast Unity dose calculation, reported in less than 40 seconds	Platform-specific implementation focused on the Unity geometry	Li et al. (34)
ArcherQA	EGSnrc-based GPU implementation	MC secondary-check engine for Unity online-adaptive plans	Approximately 1–2 minute calculation time with high 3D gamma agreement	Application-specific rather than a general-purpose MC platform	Cheng et al. (31)

CT, computed tomography; GPU, graphics processing unit; MC, Monte Carlo; QA, quality assurance; VHEE, very-high-energy electrons. This table includes both engines represented in the included studies and additional contextual examples cited to illustrate the broader technical evolution of GPU-based MC dose calculation.

For example, Liu et al. (25) reported an IMRT dose calculation time of approximately 3 seconds on an NVIDIA RTX 4080 GPU.

Early GPU-based MC engines such as GPUMCD and gPMC demonstrated roughly 50-100x speed-ups (8, 24). More recent GPU-oriented developments cited in the reviewed and contextual literature have reported accelerations ranging from approximately 1000x to 2500x, enabling complex plan doses to be calculated within seconds to minutes (25, 31, 33, 34). These advances have substantially improved the technical feasibility of MC implementation, including for time-sensitive workflows.

The underlying code lineage is also relevant when interpreting these GPU implementations. gPMC was developed as a GPU adaptation of the PENELOPE electron-photon transport framework, whereas gDPM extends transport methods derived from the DPM/EGSnrc family (35, 36). These distinctions matter because reported speed and flexibility depend not only on hardware acceleration but also on the transport model and implementation scope of the parent code.

### 3.4 AI-enhanced Monte Carlo methods

AI methods represent a complementary acceleration strategy to GPU computing. Beyond the included studies, recent contextual literature has explored AI-assisted strategies for accelerating or denoising MC dose calculations. Three main approaches have emerged: (1) neural-network denoising, which allows MC to be run with fewer particle histories and then applies a trained denoiser to recover clinical-quality dose distributions, achieving effective speedups of 100-1000x while maintaining 1-2% accuracy (9); (2) surrogate dose-prediction models that bypass particle transport entirely by using deep-learning architectures trained on MC-generated dose distributions; and (3) machine-learned beam modeling, as demonstrated by Zhao et al. (37), who used machine learning to emulate LINAC commissioning data with less than 1% error, thereby streamlining one of the most labor-intensive steps in MC deployment. These approaches are sometimes combined, for example by pairing reduced-particle GPU-MC simulations with AI denoising for final dose refinement. None of these AI-assisted MC approaches have been prospectively validated in clinical patient cohorts. Published results derive from retrospective comparisons with reference MC calculations on the beam configurations and anatomical sites used for training. Neural-network denoising is the most mature of the three approaches, but its accuracy on novel geometries, atypical patient anatomies, and different LINAC platforms has not been tested. Surrogate dose-prediction models present a further concern: because they bypass particle transport, they cannot guarantee physical consistency and may produce plausible but incorrect distributions in out-of-distribution scenarios (9). Until multi-institutional prospective validation is available, AI-assisted MC methods are research tools, not clinical replacements for physics-based MC calculation.

### 3.5 Independent QA tools versus TPS-integrated MC engines

A practical distinction in the reviewed literature is between independent MC-based QA tools and TPS-integrated MC dose engines. Independent QA tools (e.g., GPUMCD as used in Mobius3D, ArcherQA) perform secondary dose checks against the clinical TPS and serve as an independent verification layer for detecting potential planning errors (31, 38). For high-precision applications such as SRS and SBRT, refinement of the HD-MLC beam model within commercial QA systems has been shown to substantially improve small-field gamma-based agreement with reference dose distributions (19). These tools typically use their own beam models and are not involved in plan optimization. In contrast, TPS-integrated MC engines (e.g., Elekta Monaco’s XVMC) serve as the primary dose-calculation algorithm within the planning system and directly influence plan optimization and final dose distributions (10, 11). This distinction has practical implications: TPS-integrated MC improves the accuracy of the optimized plan itself, whereas independent MC QA provides a separate verification layer that can identify discrepancies arising from algorithmic limitations or planning errors. Both approaches may contribute to patient safety, and some institutions use both strategies within the same workflow.

A related methodological issue is the choice of reference distribution in gamma analysis for patient-specific QA. In MC-based TPS workflows, gamma pass rates may vary according to whether the TPS calculation, measurement set, or secondary calculation is treated as the reference, which can influence interpretation of QA agreement and the apparent impact of MC noise (32).

### 3.6 Technical considerations for clinical MC implementation

Dose-to-medium versus dose-to-water: A fundamental consideration in clinical MC implementation is the reporting convention: dose-to-medium (Dm,m) versus dose-to-water (Dw,w). MC naturally computes Dm,m, while conventional TPS algorithms typically report Dw,w. The difference is negligible in soft tissue (less than 1%) but can reach 2-4% in bone and up to 10% in dense materials such as dental implants (39). For lung tissue, differences are typically 1-2%. Since clinical experience and dose-response data have been accumulated using Dw,w from conventional algorithms, direct adoption of Dm,m could introduce systematic biases in dose prescription unless conversion factors are applied. No international consensus exists on the preferred reporting convention. The AAPM has accepted both approaches as valid (39), while the IAEA TRS-483 code of practice for small-field dosimetry assumes Dw,w for reference and relative dose determination (40). Institutions adopting MC must therefore set a local reporting policy, apply conversion factors where required, and document this choice in treatment records so that prescriptions remain consistent with the historical dose-response data on which clinical decisions are based.

Statistical uncertainty propagation in adaptive workflows: In online adaptive radiotherapy, MC dose calculations must be completed within tight time constraints (typically 2–5 minutes), which may necessitate accepting higher statistical uncertainty per fraction compared with conventional planning. Monaco’s clinical MC engine commonly runs at approximately 1% statistical uncertainty per control point for standard fractionation (41); adaptive workflows may need to accept higher per-voxel uncertainty to fit within the available time window. In stereotactic radiotherapy, the balance between dose voxel size, statistical uncertainty, and calculation time also requires optimization; Goodall and Ebert (41) showed in Monaco-based planning that clinically efficient settings could preserve acceptable agreement with high-precision reference calculations. Reducing statistical uncertainty by half requires four times the particle histories and therefore four times the computation time, a 1/√N relationship that sets the practical envelope for clinical MC deployment.

#### 3.6.1 Clinical relevance thresholds

Not all dosimetric improvements from MC necessarily translate into clinically meaningful differences. MC corrections are likely to be most relevant when they exceed 3-5% in target coverage or organ-at-risk dose, a range in which changes in tumor control probability (TCP) or normal tissue complication probability (NTCP) may become clinically important (7). Based on the reviewed evidence, scenarios in which MC corrections routinely exceeded these thresholds included: (a) small-field SRS/SBRT in lung (3-8% differences versus pencil-beam algorithms); (b) plans traversing extensive heterogeneities such as bone-tissue-air interfaces; (c) MR-Linac dosimetry, where magnetic-field effects alter electron trajectories; and (d) very small fields (less than 2 cm), where lateral electronic disequilibrium is substantial.

#### 3.6.2 Variance reduction strategies

Beyond hardware acceleration, variance reduction techniques are essential for efficient clinical MC. Common approaches include: (1) particle splitting and Russian roulette to balance computation between high- and low-importance regions; (2) photon forcing to ensure interactions in thin geometries; (3) range rejection to terminate low-energy electrons that cannot escape their current voxel; and (4) correlated sampling for comparing similar treatment plans. The GPU-MC codes reviewed here employ various combinations of these techniques, for example, GPUMCD uses interaction forcing and range rejection, while GARDEN employs adaptive splitting. The choice and tuning of variance reduction parameters represent a practical challenge for clinical implementation.

Practical implementation barriers: Several barriers remain after computational speed is no longer limiting. First, accurate MC beam modeling requires detailed accelerator-head geometry and material data, which vendors do not always disclose; users rely on published phase-space files or iterative tuning against measurements. Second, commissioning an MC beam model is more labor-intensive than configuring an analytical algorithm and requires expertise in MC transport physics that many clinical departments do not have in-house. Third, no standardized cross-platform validation protocol exists for MC dose engines. The AAPM TG-268 RECORDS checklist (16) addresses reporting, but each institution still designs its own commissioning tests, and rigor varies between centers. Fourth, regulatory frameworks in many jurisdictions have not established clear approval pathways for MC as a primary dose-calculation algorithm, which can delay adoption even when technical feasibility is demonstrated.

### 3.7 Vote counting by direction of effect

Following Cochrane Handbook Chapter 12 guidance on synthesis without meta-analysis, vote counting by direction of effect was applied to the subset of included studies that directly compared Monte Carlo-based dose calculations with another dose-calculation approach, independent measurements, TPS calculations, or reference results. Nine of the 17 included studies met this criterion (10, 11, 20, 22, 23, 26–28, 31). Across this comparative subset, all 9 studies (100%) reported MC performance that was either superior to or in agreement with the comparator/reference, and no study reported the comparator as more accurate than MC in the tested scenario. One study (20) reported clear superiority of MC over a conventional pencil-beam algorithm in a clinically important SRS setting. The remaining eight comparative studies supported MC validity through close agreement with independent measurements, TPS results, or reference calculations. Studies focused primarily on acceleration, uncertainty propagation, correction-factor derivation, or gamma-reference methodology were not included in this direction-of-effect denominator because they did not provide a directly comparable superiority/equivalence judgment.

### 3.8 Study quality and vendor distribution

Overall, the evidence base in this technical domain was judged to be moderate to high in quality. More than half of the included studies were rated as high quality. It should also be noted that none of the reviewed studies directly measured clinical outcomes such as tumor control or toxicity. The reported gains are therefore dosimetric, and although radiobiological models suggest that 3-5% improvements in dose accuracy could potentially influence clinical outcomes (7), this remains a model-based inference that requires prospective validation. Lower-quality studies still contributed contextual information, but they had greater methodological limitations. Importantly, none of those lower-quality studies contradicted the direction of findings reported by the higher-quality studies.

## 4 Discussion

### 4.1 Synthesis of findings

This review shows that Monte Carlo dose calculation is now clinically applicable, and that the magnitude of its clinical value depends on the treatment scenario. MC accuracy is well established; the more useful question for current practice is in which clinical situations that accuracy is large enough to change patient management.

Three themes emerged from the synthesis. First, MC accuracy gains were clinically consequential in identifiable high-risk scenarios (lung SBRT, small-field SRS, MR-Linac dosimetry) and clinically marginal in routine homogeneous-site treatments. Second, GPU acceleration has removed the computational barrier; the remaining bottleneck is institutional, including commissioning, training, and regulatory factors. Third, implementation pathways differed by vendor: Elekta offers TPS-integrated MC in Monaco, whereas Varian-associated workflows more often used MC for independent quality assurance.

### 4.2 Accuracy gain and clinical relevance

**Table 4** summarises, by clinical scenario, the conventional-algorithm limitation, the expected magnitude of the Monte Carlo correction, whether Monte Carlo is likely to change the clinical decision, and the recommended action.

**Table 4:** Clinical decision matrix: when Monte Carlo is likely to change the clinical plan.

Clinical scenario	Conventional algorithm limitation	MC correction magnitude	Would MC change the clinical decision?	Recommended action
Lung SBRT (peripheral tumors near chest wall)	Pencil-beam: 3-8% error at tissue-air interfaces	3-8% correction in PTV D95	Yes; may require dose escalation or replanning to maintain target coverage	Use MC as primary dose engine or mandatory independent QA check
Small-field SRS (<2 cm targets)	Lateral electronic disequilibrium not modeled	2-5% output factor correction	Yes; output factor errors directly affect delivered dose to small targets	Independent MC verification before treatment delivery
MR-Linac (1.5 T magnetic field)	Electron return effect not modeled by conventional algorithms	1-3% dose redistribution at tissue-air boundaries	Yes; magnetic-field effects are systematic	TPS-integrated MC (Monaco) required for accurate planning
Standard IMRT in homogeneous sites (e.g., pelvis, brain without implants)	Acuros XB or collapsed-cone: within 1-2% of MC	1-2%	Unlikely; difference below clinical relevance threshold	MC not routinely required; conventional algorithms adequate
Head and neck IMRT with dental implants	Dm,m vs Dw,w divergence: up to 10% near dense materials	Material-dependent, potentially >5% locally	Case-dependent; clinically significant only near high-density interfaces	Evaluate on a case-by-case basis; MC advisable when OAR constraints are tight near implants
Complex heterogeneity (bone-air-tissue interfaces)	Superposition/convolution: 2-4% error at interfaces	2-4% at boundaries	Case-dependent; significant when target or critical OAR lies at the interface	Independent QA check recommended for plans with extensive heterogeneous path lengths

All included studies showed that MC reduces dosimetric uncertainty in situations where conventional algorithms perform less well. A dosimetric improvement does not automatically translate into a clinical improvement. The relevant distinction for practice is between scenarios in which MC corrections are large enough to change the treatment plan and scenarios in which the correction is measurable but clinically inconsequential.

Clinically consequential scenarios (MC corrections >3-5%). Using the 3-5% threshold at which TCP and NTCP changes become clinically important (7), three scenarios from the reviewed evidence are clearly consequential. In lung SBRT, MC corrections of 3-8% relative to pencil-beam algorithms (20) alter PTV D95 coverage and can trigger dose escalation or replanning. For a moderate hypofractionation regimen such as 48 Gy in 4 fractions, the nominal prescription gives a biologically effective dose (BED, α/β = 10) of approximately 106 Gy, close to the 100 Gy threshold associated with higher local control in lung SBRT (42); a 5% dose reduction at the tumor-lung interface would drop the BED below this threshold, eroding the dose margin relevant for local tumor control. In MR-Linac dosimetry, the electron return effect at tissue-air boundaries is not modeled by conventional algorithms and produces 1-3% dose redistribution that is systematic across fractions in non-adaptive workflows. In online adaptive workflows each fraction’s plan accounts for the effect independently, but the underlying physics still requires MC-level modeling for accurate dose prediction (28, 29). In small-field SRS (<2 cm), output factor corrections of 2-5% affect the absolute dose delivered to small targets, where the steep dose-response gradient makes even modest errors clinically relevant (27).

Clinically marginal scenarios (MC corrections <2%). In homogeneous treatment sites with standard field sizes, the difference between MC and well-commissioned conventional algorithms such as Acuros XB or collapsed-cone convolution was consistently within 1-2% (22, 23). For a pelvic IMRT plan where MC changes organ-at-risk dose by 1%, the plan typically proceeds unchanged because this difference falls within the overall dose delivery uncertainty of the radiotherapy chain, estimated at approximately 3.5% (one standard deviation) when all sources are combined (43). For brain treatments without metallic implants, well-validated analytical algorithms also provide accuracy sufficient for routine planning.

Implications for resource allocation. MC implementation should be prioritized for the scenarios identified above rather than applied indiscriminately across all clinical workflows. Institutions with limited resources can adopt a tiered approach: using MC for high-risk scenarios (lung SBRT, SRS, MR-Linac) and continuing to use conventional algorithms for routine homogeneous-site treatments. This is relevant for centers expanding linac-based SRS to complex indications such as multiple brain metastases, where conformity, gradient control, and normal-brain sparing are the main planning priorities (44).

### 4.3 Clinical feasibility and workflow integration

As described in Section 3.3, GPU acceleration has removed computation time as the main barrier to clinical MC adoption. The remaining barriers are institutional: beam model commissioning, staff expertise, regulatory approval, and Dm,m/Dw,w policy. Elekta’s Monaco system shows that TPS-integrated MC is technically mature. Cheng et al. (31) reported that GPU-MC verification can be completed within the 2–5 minute clinical window required for online adaptive therapy, with per-fraction statistical noise partially averaging out over the treatment course. Broader integration across non-Elekta platforms will depend on vendor willingness to incorporate MC as a primary dose engine or to support third-party MC integration through standardized data interfaces.

### 4.4 Vendor-specific implementation landscape

The vendor distribution in this review should be interpreted cautiously and should not be taken to imply platform superiority. Elekta-associated studies formed the largest subgroup (47.1%), which partly reflects two structural factors: Monaco is the only widely used commercial TPS with a native clinical MC dose engine, and the Unity MR-Linac has generated substantial MC research because of the dosimetric challenges introduced by its 1.5 T magnetic field. Varian’s Eclipse platform does not currently offer a native MC dose engine. Varian-associated MC studies therefore used external MC codes for independent verification, commissioning, or algorithm comparison rather than evaluating TPS-integrated MC planning (20, 22, 23). Siemens-specific evidence was limited to one Artiste validation study (26), reflecting Siemens’s exit from the LINAC market.

This concentration has implications for the generalizability of the review’s conclusions. Findings about MC dosimetric accuracy in heterogeneous media, small fields, and magnetic-field environments reflect transport physics and apply across platforms. Findings about TPS-integrated MC feasibility are specific to the Elekta/Monaco ecosystem and cannot be directly extrapolated to Varian or other vendor workflows without equivalent integration. The review’s conclusion that MC is clinically feasible therefore applies to Monaco-based workflows in current commercial form, while remaining technically achievable but not yet implemented for other major platforms.

### 4.5 Subgroup observations

Four subgroup observations emerged from the analysis: (1) TPS-integrated MC in Monaco and independent MC-based QA tools address different stages of the workflow; the former improves the optimized plan itself, whereas the latter adds an external verification layer. (2) MR-Linac studies showed MC to be particularly important because magnetic-field effects are not modeled adequately by conventional algorithms. (3) GPU-accelerated studies consistently reported large speedups relative to CPU-based MC implementations. (4) Findings from high-quality studies were broadly consistent with those from moderate-quality studies, supporting the overall coherence of the evidence base.

### 4.6 Remaining challenges

Several practical requirements remain. The dose-to-medium versus dose-to-water reporting convention requires institutional consensus, because much of the historical dose-response literature was derived using conventional Dw,w-equivalent approaches. User training is also essential; medical physicists and dosimetrists must be familiar with MC-specific concepts such as statistical uncertainty, variance reduction, and workflow differences. Greater vendor cooperation in providing detailed accelerator-head geometry and material data would facilitate accurate MC model development. Regulatory pathways for MC as a primary dose-calculation algorithm also require clarification. Finally, the absence of direct clinical outcome data comparing MC-planned and conventionally planned treatments remains an important evidence gap. Prospective studies linking MC-based planning to measurable differences in tumor control or toxicity would strengthen the case for broader adoption.

### 4.7 Limitations of this review

Several limitations should be acknowledged. First, the vendor-specific search terms may have excluded relevant studies, particularly those developing vendor-agnostic MC methods or validating platforms that were not explicitly named. Manual reference-list screening partially mitigated but could not eliminate this limitation. Second, no formal risk-of-bias instrument validated for technical simulation studies was available; the adapted quality framework was predefined and applied independently by two reviewers, but it has not undergone external validation. Third, publication bias likely favors successful MC implementations, because negative or null computational dosimetry findings are less frequently reported. Fourth, the rapid pace of development in GPU computing and AI means that studies published after the September 2025 search cutoff may already have introduced advances not captured here. These limitations should be considered when interpreting the review, although the findings were broadly consistent across studies and platforms.

## 5 Conclusion

First, MC dose calculation should be prioritized for clinical scenarios in which dosimetric corrections routinely exceed 3-5%: lung SBRT at tissue-air interfaces, small-field SRS with targets smaller than 2 cm, and MR-Linac treatments where the magnetic field alters electron trajectories at tissue-air boundaries. In these scenarios MC corrections are large enough to affect target coverage or organ-at-risk doses in ways that change clinical decisions. For routine treatments in homogeneous sites with standard field sizes, well-commissioned conventional algorithms such as Acuros XB or collapsed-cone convolution provide adequate accuracy, and universal MC adoption is not supported by the current evidence.

Second, GPU acceleration has resolved the computational barrier that historically limited clinical MC adoption. The remaining barriers are institutional: beam model commissioning requires specialized expertise and vendor cooperation, no standardized cross-platform validation protocol exists, regulatory pathways for MC as a primary dose engine are unclear in many jurisdictions, and institutions must set explicit Dm,m versus Dw,w reporting policies before clinical deployment.

Third, the evidence base for MC in radiotherapy is dosimetric. No included study measured clinical endpoints such as tumor control or normal tissue complication rates. Prospective studies comparing clinical outcomes between MC-planned and conventionally planned treatments are the most important evidence gap and should be a priority for the field.

Fourth, institutions considering MC adoption should: (a) define a local Dm,m/Dw,w reporting policy with documented conversion factors; (b) set acceptable statistical uncertainty tolerances for different clinical scenarios, using published benchmarks of approximately 1% for standard planning and higher values where time-constrained adaptive workflows require a trade-off (41); (c) commission MC beam models using a systematic protocol that includes independent measurement verification; and (d) ensure that staff involved in MC-based planning are trained in MC transport physics, uncertainty interpretation, and variance reduction parameter selection.

## Data Availability

The original contributions presented in the study are included in the article/supplementary material. Further inquiries can be directed to the corresponding author.
